# Potential therapeutic effect of targeting glycogen synthase kinase 3β in esophageal squamous cell carcinoma

**DOI:** 10.1038/s41598-020-68713-9

**Published:** 2020-07-16

**Authors:** Dilireba Bolidong, Takahiro Domoto, Masahiro Uehara, Hemragul Sabit, Tomoyuki Okumura, Yoshio Endo, Mitsutoshi Nakada, Itasu Ninomiya, Tomoharu Miyashita, Richard W. Wong, Toshinari Minamoto

**Affiliations:** 1 Division of Translational and Clinical Oncology, Cancer Research Institute, Kanazawa University, 13-1 Takara-machi, Kanazawa, 920-0934 Japan; 2 Central Research Resource Branch, Cancer Research Institute, Kanazawa University, Kanazawa, Japan; 3 Department of Neurosurgery, Graduate School of Medical Sciences, Kanazawa University, Kanazawa, Japan; 4 Department of Gastroenterological Surgery, Graduate School of Medical Sciences, Kanazawa University, Kanazawa, Japan; 5 WPI Nano Life Science Institute, Kanazawa University, Kanazawa, Japan; 6 Department of Surgery and Science, Graduate School of Medicine and Pharmaceutical Sciences, University of Toyama, Toyama, Japan; 7 Department of Surgical Oncology, Kanazawa Medical University Hospital, Ishikawa, Japan

**Keywords:** Cancer, Cell biology

## Abstract

Esophageal squamous cell carcinoma (ESCC) is a common gastrointestinal cancer and is often refractory to current therapies. Development of efficient therapeutic strategies against ESCC presents a major challenge. Glycogen synthase kinase (GSK)3β has emerged as a multipotent therapeutic target in various diseases including cancer. Here we investigated the biology and pathological role of GSK3β in ESCC and explored the therapeutic effects of its inhibition. The expression of GSK3β and tyrosine (Y)216 phosphorylation-dependent activity was higher in human ESCC cell lines and primary tumors than untransformed esophageal squamous TYNEK-3 cells from an ESCC patient and tumor-adjacent normal esophageal mucosa. GSK3β-specific inhibitors and small interfering (si)RNA-mediated knockdown of GSK3β attenuated tumor cell survival and proliferation, while inducing apoptosis in ESCC cells and their xenograft tumors in mice. GSK3β inhibition spared TYNEK-3 cells and the vital organs of mice. The therapeutic effect of GSK3β inhibition in tumor cells was associated with G0/G1- and G2/M-phase cell cycle arrest, decreased expression of cyclin D1 and cyclin-dependent kinase (CDK)4 and increased expression of cyclin B1. These results suggest the tumor-promoting role of GSK3β is via cyclin D1/CDK4-mediated cell cycle progression. Consequently, our study provides a biological rationale for GSK3β as a potential therapeutic target in ESCC.

## Introduction

Esophageal cancer is one of the most common causes of cancer-related death worldwide and is thus a major health challenge. Esophageal squamous cell carcinoma (ESCC) accounts for nearly 90% of all esophageal cancer cases globally and is highly prevalent in East Africa and Asia, including China and Japan^1,2^. Most patients with advanced stage ESCC require intensive treatment including chemotherapy, chemo-radiotherapy, surgery and combinations of these^3^. However, many patients are refractory to such multi-disciplinary treatments, thereby resulting in dismal clinical outcomes^4,5^. During the last two decades, an increasing number of biologically targeted agents against growth factors (e.g., HER2, epidermal growth factor receptor), angiogenic factors (e.g., vascular endothelial growth factors) and molecules involved in the immune checkpoint have been developed. Some have been approved for the treatment of various cancer types, including esophageal cancer^6–8^. However, ESCC patients appear to derive little benefit from these agents^9,10^. There is still insufficient knowledge of the biological basis of ESCC, despite the whole genome studies of ESCC^11–13^. Therefore, identification of new therapeutic targets is of paramount importance to combat refractory ESCC^10^.

Accumulating studies indicate that esophageal squamous cell carcinogenesis is initiated by a combination of extrinsic/environmental (e.g., alcohol, cigarette smoking) and intrinsic (e.g., acetaldehyde, an intermediate metabolite of alcohol) carcinogenic factors^2,4,5^. These carcinogenic stimuli cause ESCC to develop through a sequential transformation of squamous epithelial cells to squamous dysplasia (mild to severe, or low-grade and high-grade intraepithelial neoplasia), intraepithelial (in situ) SCC, and ultimately progressing to invasive SCC^14^. An early biochemical characteristic in the process of ESCC development is the lack of glycogen associated with cellular neoplastic transformation. This characteristic enables Lugol’s iodine-dye endoscopy, which has long been used for screening and diagnosis of ESCC^2,5^. Loss of cellular glycogen occurs in mild squamous dysplasia (or low-grade intraepithelial neoplasia) and persists till invasive ESCC^2^, suggesting that impaired glycogen synthesis and/or excess glycogenolysis is involved in ESCC development and progression rather than being a consequence of cellular neoplastic transformation. Glycogen synthase (GS) responsible for glycogenesis is the primary substrate of glycogen synthase kinase (GSK)3β and is inactivated by phosphorylation of its serine (S) 641 residue by GSK3β^15,16^. Thus, we hypothesized that GSK3β is aberrantly expressed and/or activated in ESCC.

GSK3β was initially identified as a serine/threonine protein kinase that phosphorylates and inactivates GS. Subsequent studies revealed that GSK3β regulates multiple biological pathways to maintain normal cellular life and homeostasis^15,16^. GSK3β is normally inactive in cells when its S9 residue is phosphorylated. Upon turning from the inactive to active form following phosphorylation of its tyrosine (Y) 216 residue, GSK3β has been shown to participate in various diseases including glucose intolerance, neurodegenerative disorders, and chronic inflammatory and immunological diseases^17^. Despite its functions against some pro-oncogenic pathways in untransformed cells^18^, we have shown that aberrant expression and activity of GSK3β in tumors sustains tumor cell survival and proliferation as well as invasion and therapy resistance in gastrointestinal and pancreatic cancer, glioblastoma and bone and soft tissue sarcomas. We have also previously clarified the pathways underlying the tumor-promoting roles of GSK3β, as well as the therapeutic effects of GSK3β inhibition against these cancer types^19,20^. Several other studies together with ours have advocated GSK3β as a potential theranostic target in more than 25 different cancer types^19–23^. These include rare cancer types such as neuroendocrine neoplasms in the gastrointestinal tract and pancreas^24,25^. Building upon this background knowledge, in the present study we investigated the putative pathological roles for GSK3β in ESCC and explored the effects of GSK3β inhibition against this tumor type and the underlying biological mechanisms.

## Materials and methods

### Cell lines and ESCC patients

Human ESCC cell lines (TE-1, TE-5, TE-8, TE-9, TE-10 and TE-15) were obtained from the American Type Culture Collection. We previously established human ESCC KES cell line^26^. These cell lines were maintained in RPMI medium (Sigma-Aldrich) supplemented with 10% fetal bovine serum and 100 U/mL penicillin–streptomycin at 37 °C with 5% CO_2_. We prepared normal squamous epithelial TYNEK-3 cells from non-neoplastic esophageal squamous mucosa obtained from the surgical specimen of an ESCC patient, as described previously^27^. The cells were maintained in Keratinocyte SFM (Thermo Fisher Scientific).

This study included 46 patients who underwent surgery for ESCC in the Department of Surgical Oncology at Kanazawa University Hospital between 1988 and 2006 and provided informed consent at the time of the initial diagnosis in compliance with guidelines of Kanazawa University and in accordance with Declaration of Helsinki. Surgical specimens were fixed in neutralized 10% formalin and embedded in paraffin for routine histopathologic examination and immunohistochemical analysis as described below. Histopathologic characteristics and tumor stage at surgery were defined according to WHO Classification of Tumors of the Digestive System^14^ and the TNM classification^28^, respectively. The Kanazawa University Medical Ethics Committee and the University of Toyama Institutional Review Board approved the design and protocols including all experiments in this study.

### Western blotting

Cellular protein was extracted from cultured cells and fresh xenograft tumor specimens using lysis buffer (CelLytic MT; Sigma-Aldrich) containing a mixture of protease and phosphatase inhibitors (Sigma-Aldrich). A 20 μg-aliquot of protein extract was analyzed by Western blotting for the proteins of interest^29^. The amount of protein in each sample was monitored by expression of β-actin. The following primary antibodies were used at the dilutions shown against both GSK3 isoforms (GSK3α and GSK3β; 1:1,000; Millipore), GSK3β (1:1,000; BD Biosciences) and GSK3β fractions that are phosphorylated at the serine (S) 9 residue (pGSK3β^S9^; 1:1,000; Cell Signaling Technology) and the tyrosine (Y) 216 residue (pGSK3β^Y216^; 1:1,000; BD Biosciences); glycogen synthase (GS; 1:1,000; Cell Signaling Technology) and its fraction phosphorylated at the S641 residue (pGS^S641^; 1:1,000; Cell Signaling Technology); cyclin D1 (1:1,000; MBL); cyclin-dependent kinase (CDK)4 (1:1,000; Abcam), cyclin B1 (1:1,000, Cell Signaling Technology), poly[ADP]-ribose polymerase (PARP; 1:1,000; Cell Signaling Technology), cleaved (c)-PARP (1:1,000; Cell Signaling Technology), and β-actin (1:4,000; Ambion).

### Measurement of intracellular glycogen

Concentration of intracellular glycogen was analysed using Glycogen Colorimetric Assay Kit II (BioVision) and was compared between TYNEK-3 and ESCC cell lines. TE-5, TE-8 and TE-10 cells were treated for 24 h with dimethyl sulfoxide (DMSO; Sigma-Aldrich) or with one of the GSK3β inhibitors: AR-A014418 (Calbiochem)^30^ or SB-216763 (Sigma-Aldrich)^31^ at 25 μmol/L. Then, intracellular glycogen concentrations were compared between the respective cells treated with DMSO or either of the GSK3β inhibitors.

### Immunohistochemical examination

Representative paraffin sections of primary tumors and corresponding adjacent non-neoplastic (at the proximal surgical margin) tissues from ESCC patients were examined for expression and phosphorylation of GSK3β and GS by the avidin–biotin-peroxidase complex (ABC) method as described previously^32^. Tissue sections were deparaffinized and then microwaved for 15 min in Target Retrieval Solution (pH 9.0; Dako). Nonspecific immune reaction was blocked by incubation of sections in methanol containing 0.3% H_2_O_2_ for 30 min followed by incubation in 5% skim milk (Wako) at room temperature for 1 h. The sections were incubated with either of the mouse antibodies against GSK3β (1:200 dilution) and pGSK3β^Y216^ (1:500) (both from BD Biosciences) or of the rabbit antibodies against GS (1:200) and pGS^S641^ (1:200) (both from Cell Signaling Technology) overnight at 4 °C. They were then washed and the corresponding secondary antibody was applied for 30 min. Sections were exposed to diaminobenzidine peroxidase substrate (Funakoshi) for 1 min and counterstained with Mayer’s hematoxylin. Histological and immunohistochemical images were observed and captured using Keyence BZ-X700 Analyzer (Version 1.3, Keyence). For the respective molecules, the mean percentage of immunohistochemistry (IHC)-positive tumor cells in 5 microscopic fields was calculated for each tumor section and classified into five scores as follows: 0, < 10%; 1, 10 ~ 25%; 2, 26 ~ 50%; 3, 51 ~ 75%; and 4, > 76%. Based on the IHC scores, the expression level of the respective molecules was determined as low (score 0 to 2) or high (score 3 and 4) according to our previous study^32^.

### ESCC databases analysis

Genomic and molecular profiles of ESCC were obtained from The Cancer Genome Atlas (TCGA; https://cancergenome.nih.gov/). According to the previous report^33^, the analysis tool UALCAN (https://ualcan.path.uab.edu/) was used to study levels of GSK3β mRNA expression in normal (n = 11) and tumor (n = 184) tissues in ESCC patients, and to compare the levels of tumor GSK3β mRNA expression with different clinicopathologic features including age, gender, smoking habits and body weight of patients, and with tumor grades and stages.

### Analyses for cell survival, proliferation and apoptosis

Cells seeded in 96-well plates were treated with DMSO or with one of the GSK3β inhibitors: AR-A014418^30^, SB-216763^31^ or LY2090314 (Sigma-Aldrich)^34^ dissolved in DMSO at the indicated final concentration in the medium. The concentrations of GSK3β inhibitors used in this study are within the range of pharmacologically relevant doses, as previously reported^30,31,34^. At designated time points, the relative numbers of viable cells were determined using the WST-8 (4-[3-(4-iodophenyl)-2-(4-nitrophenyl)-2H-5-tetrazolio]-1,3-benzene disulfonate) assay kit (Cell Counting Kit-8; Dojindo). After treatment with DMSO or GSK3β inhibitor, the relative numbers of proliferating and apoptotic cells were determined using the Click-iT Plus 5-ethynyl-2′-deoxyuridine (EdU) Alexa Fluor 555 Imaging Kit (Thermo Fisher Scientific) and the Cellular DNA Fragmentation ELISA kit (Roche Diagnostics), respectively. Using fluorescence microscopy (Keyence BZ-X700 Analyzer, Version 1.3), proliferating cells positive for EdU in nuclei were scored following treatment with DMSO or with one of the GSK3β inhibitors. The mean percentage of cells positive for nuclear EdU in 5 microscopic fields was calculated with standard deviations (SDs). The occurrence of apoptosis was further shown by observing changes in cell-cycle fractions as described below.

### RNA interference (RNAi)

Small interfering RNA (siRNA) specific to human GSK3β (GSK3β Validated Stealth RNAi) and negative control siRNA (Stealth RNAi Negative Control Low GC duplex) were purchased from Invitrogen. The specificity of GSK3β-specific siRNA was confirmed in our previous studies^29,35^. Cells were transfected with 20 nmol/L of either siRNA using Lipofectamine RNAiMAX (Invitrogen). The effect of RNAi on GSK3β expression was determined by Western blotting using an antibody that recognizes both GSK3α and GSK3β (Millipore). To examine the effect of GSK3β RNAi on cell survival, proliferation and apoptosis, cells were transfected with 20 nmol/L of control or GSK3β-specific siRNA. At 72 h after transfection, the relative numbers of viable, proliferating and apoptotic cells were measured as described above.

### Analysis of cell cycle profile

The cell cycle profile was analyzed by fluorescence-activated cell sorting (FACS) as described previously^36^. Briefly, ESCC cells were treated with DMSO, 25 μmol/L AR-A014418 or SB-216763 for 48 h, or transfected with negative control siRNA or GSK3β-specific siRNA for 72 h. The cells were then trypsinized, washed twice with phosphate buffered saline (PBS), and fixed in 70% ethanol at – 20 °C overnight. Fixed cells were suspended in PBS containing 50 μg/mL RNase A (Nacalai Tesque) and 50 μg/mL propidium iodide (PI) (Sigma-Aldrich). Cellular DNA content was analyzed using a FACS Canto II with FACSDiva software (Version 8.0, BD Bioscience).

### Animal study

The effect of GSK3β inhibition on tumor proliferation was examined on ESCC TE-8 cell xenografts in athymic mice. A total of 1 × 10^6^ TE-8 cells suspended in 50 μL of PBS were subcutaneously inoculated into each of 25 BALB/c athymic mice (Charles River Laboratories, Japan). Mice were randomly assigned to five groups and given tri-weekly intraperitoneal (i.p.) injections of DMSO or of the GSK3β inhibitors AR-A014418 at different doses (2 mg/kg and 5 mg/kg body weight) or LY2090314 (1 mg/kg and 2.5 mg/kg body weight) for 5 weeks. Assuming that total body fluid in mice accounts for about 60% of their body weight, AR-A014418 doses of 2 mg/kg and 5 mg/kg body weight correspond to concentrations of approximately 10 μmol/L and 25 μmol/L in culture media respectively^29^, and LY2090314 doses of 1 mg/kg and 2.5 mg/kg body weight correspond to concentrations of approximately 2 μmol/L and 5 μmol/L, respectively. These are known pharmacological doses for these agents^30,34^. Throughout the experiment, all mice were carefully observed each day for adverse events and their body weight and tumors (in two dimensions) were measured twice a week. Tumor volume (cm^3^) was calculated using the formula: 0.5 × S^2^ × L, where S is the smallest tumor diameter (cm) and L is the largest (cm)^29^. The design and protocol of animal experiment and changes in body weights of animals during treatment are shown in the Supplementary Information, Fig. S1. All animal experiments were undertaken according to Japanese animal ethics guidelines^37^. The protocol was approved by the Institute for Experimental Animal Work, Kanazawa University Advanced Science Research Center.

At necropsy, tumors were removed and divided into three parts for fresh frozen storage, fixed in 10% neutral-buffered formalin or fixed in 4% paraformaldehyde and paraffin embedded for biochemical, histopathologic and immunohistochemical/immunofluorescence staining, respectively. Expression levels for GSK3β, cyclin D1 and pGSK3β^Y216^ were evaluated by Western blotting for protein extracts from frozen tumor specimens as described above. Paraffin sections of the tumors were stained with HE for histopathologic examination by a certified pathologist (H.S.). Representative sections of the tumors were immunostained with antibodies against GSK3β (diluted 1:500; Cell Signaling Technology), pGSK3β^Y216^ (diluted 1:500; Cell Signaling Technology), GS (diluted 1:200, Cell Signaling Technology), pGS^S641^ (diluted 1:200, Cell Signaling Technology), cyclin D1 (diluted 1:500; Cell Signaling Technology) and Ki-67 (diluted 1:700; Thermo Fisher Scientific) by the ABC method as described above. Apoptosis was detected in tumor xenografts by the terminal deoxynucleotidyl transferase-mediated dUTP nick end labeling (TUNEL) method using the In Situ Apoptosis Detection TUNEL kit (Takara). The stained tissue sections were observed using Keyence BZ-X700 Analyzer (Version 1.3). Tumor cells from 5 microscopic fields were scored for GSK3β, pGSK3β^Y216^, GS, pGS^S641^, cyclin D1 or TUNEL. The mean percentages of positive cells were calculated with SDs and compared between the tumors of mice treated with DMSO and GSK3β inhibitors at different doses. Apoptosis in tumors was further evaluated by immunofluorescence staining of paraformaldehyde-fixed tumor sections with antibodies against PARP (1:500, Cell Signaling Technology) and c-PARP (1:500, Cell Signaling Technology) followed by nuclear staining with 4′,6-diamidino-2-phenylindole (DAPI; H-1200, Vector Laboratories). Immunofluorescence images were observed and captured using Keyence BZ-X700 Analyzer (Version 1.3).

### Statistical analysis

The Student's t test was used to determine statistical differences for the data, with a *P* value of < 0.05 considered to be statistically significant. IHC scores between normal tissues and tumors of ESCC patients were statistically analyzed by One-way ANOVA test using GraphPad Prism 5.0 (GraphPad Software, Inc. CA) and compared with clinical and pathologic characteristics by Chi-square test.

## Results

### Expression and phosphorylation-dependent activity of GSK3β in ESCC cells and patient tumors

**Figure 1:**
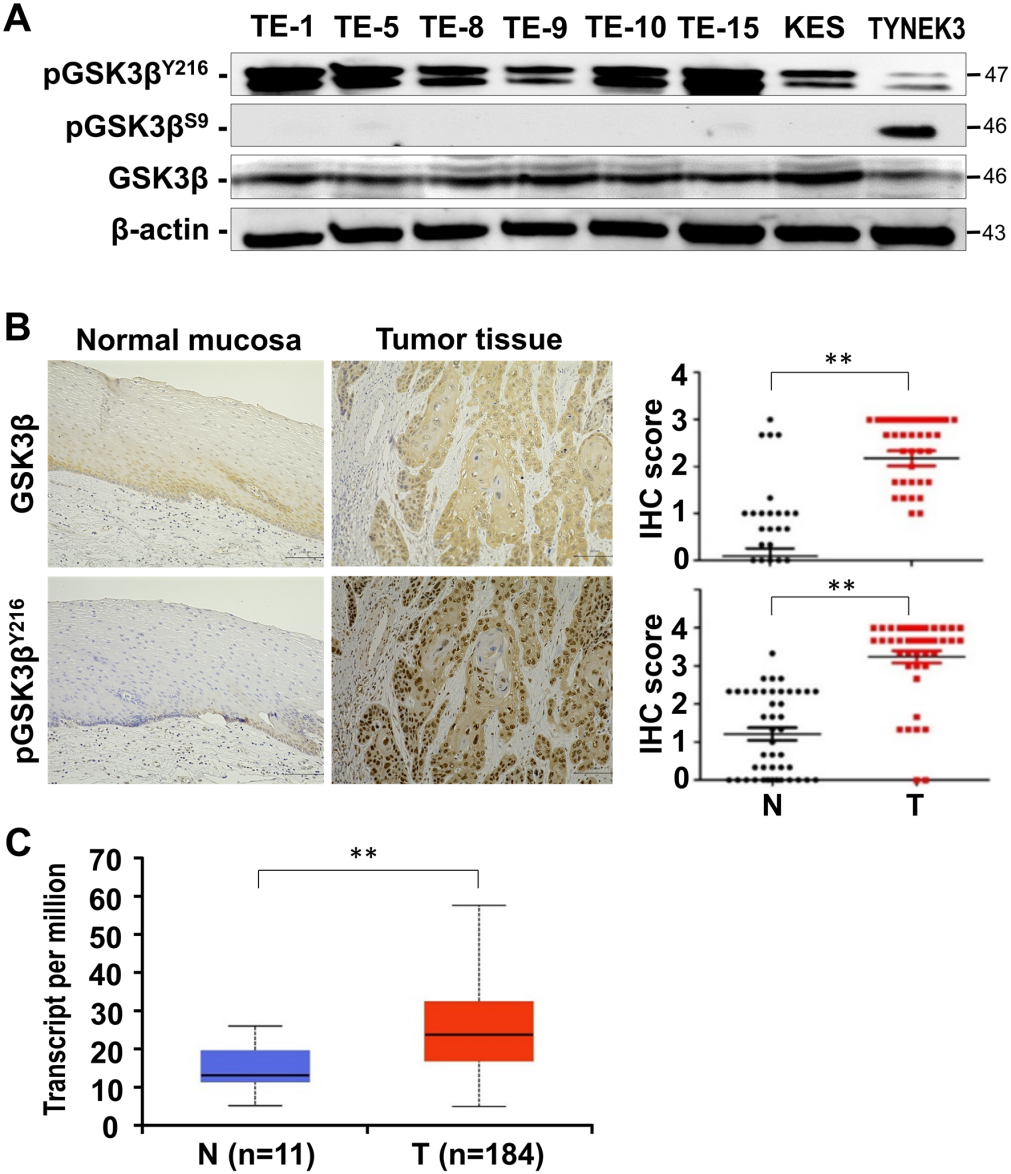
Comparative analysis for the expression and phosphorylation of GSK3β in human ESCC cells (TE-1, TE-5, TE-8, TE-9, TE-10, TE-15, KES), normal esophageal squamous epithelial cells (TYNEK-3), and normal squamous mucosa and primary tumors from ESCC patients. (**A**) Expression of GSK3β and of its phosphorylated forms (pGSK3β^S9^, inactive form; pGSK3β^Y216^, active form) were examined by Western blotting. β-actin expression was monitored as a loading control in each sample. (**B**) Representative findings for the expression of GSK3β and its Y216 phosphorylated fraction (pGSK3β^Y216^) in the primary tumor and corresponding normal squamous mucosa of ESCC patients. The scale bar indicates 100 μm in length. Immunohistochemical images were captured using Keyence BZ-X700 Analyzer (Version 1.3). The two right hand graphs generated using GraphPad Prism 5.0 (GraphPad Software, Inc. CA) show statistical comparison of the immunohistochemistry (IHC) scores for GSK3β and pGSK3β^Y216^ between the primary tumor (T) and normal mucosa (N) of ESCC patients. A horizontal bar in each group shows the mean value of IHC scores. **(C)** Expression of GSK3β mRNA in normal esophageal tissues (N) and primary ESCC tumor tissues (T) based on the TCGA database. The data was generated using the analysis tool UALCAN (https://ualcan.path.uab.edu/)^33^. n, number of patients; **P < 0.01. Full-length blots for (**A**) are shown in Supplementary Information, Fig. S9.

The expression levels of GSK3β and its Y216 phosphorylated fraction (pGSK3β^Y216^, active form) were higher in all ESCC cell lines compared to normal esophageal squamous TYNEK-3 cells (Fig. 1A), with less detectable S9 phosphorylation (pGSK3β^S9^, inactive form). Increased expression and activity of GSK3β in ESCC cells was also supported by the finding that S641 phosphorylation of GS (pGS^S641^, inactive form), the primary substrate of GSK3β^15,16^, was higher in ESCC than in TYNEK-3 cells (Supplementary Information, Fig. S2A). The levels of intracellular glycogen in ESCC cell lines were significantly lower than normal TYNEK-3 cells and were restored following treatment with GSK3β inhibitors (Supplementary Information, Fig. S2B).

We next examined the expression and phosphorylation of these enzymes in primary tumors and corresponding normal squamous mucosa from 46 ESCC patients using IHC. GSK3β expression and levels of pGSK3β^Y216^ and pGS^S641^ in the primary tumors were increased in most cases compared with normal squamous mucosa. IHC scores were significantly higher in the tumors than in normal mucosa (Fig. 1B, Supplementary Information, Fig. S2C). By comparing with clinical and pathologic characteristics, tumor GSK3β expression was significantly correlated with venous invasion of tumor cells and with the presence of lymph node metastasis. Significant association was also found between the level of pGSK3β^Y216^ and the presence of lymph node metastasis (Table 1). Levels of GSK3β and pGSK3β^Y216^ in the tumors tended to be associated with advanced tumor stages (III and IV), although this did not reach statistical significance (*P* = 0.22; Table 1). Similar trends were observed for the levels of GSK3β mRNA in normal and tumor tissues of ESCC patients referenced from the TCGA database (Fig. 1C, Supplementary Information, Fig. S3). Together, the results obtained with the ESCC cells and primary tumors suggest that deregulated expression and activity of GSK3β is a characteristic of ESCC and facilitates its progression.

**Table 1:** Comparison of the levels of expression and phosphorylation of GSK3β and GS in the primary tumors with the clinicopathologic parameters of ESCC patients.

Parameters	GSK3β			pGSK3β^Y216^			GS			pGS^S641^		
	Low	High	*P*-value	Low	High	*P*-value	Low	High	*P*-value	Low	High	*P*-value
**Gender**												
Female	4	5	*P* = 0.634	7	2	*P* = 0.838	1	8	*P* = 0.447	4	5	*P* = 0.236
Male	11	26		27	10		5	32		7	30	
**Age (years)**												
< 60	6	15	*P* = 0.797	12	9	*P* = 0.176	6	15	*P* = 0.056	6	15	*P* = 0.571
≥ 60	7	19		20	8		1	24		5	23	
**Histology**												
WD	3	13	*P* = 0.65	11	5	*P* = 0.83	2	14	*P* = 0.75	3	13	*P* = 0.84
MD	4	14		12	6		4	16		3	15	
PD	4	8		7	5		3	10		3	9	
**Venous invasion**												
Absent	9	13	*P* = 0.024	3	19	*P* = 0.296	1	21	*P* = 0.259	1	21	*P* = 0.496
Present	2	22		5	19		2	22		1	23	
**LN metastasis**												
Absent	9	11	*P* = 0.009	8	12	*P* = 0.047	10	10	*P* = 0.026	9	11	*P* = 0.05
Present	2	24		3	23		4	22		6	20	
**Stage (TMN)**												
I	5	7	*P* = 0.479	3	9	*P* = 0.722	4	8	*P* = 0.23	3	9	*P* = 0.92
II	4	12		2	14		1	15		3	13	
III	1	7		2	6		1	7		1	7	
IV	2	8		3	7		1	9		2	8	
I + II	9	19	*P* = 0.401	5	25	*P* = 0.195	5	23	*P* = 0.798	6	22	*P* = 0.84
III** + **IV	3	15		5	13		2	16		3	15	

*GS* glycogen synthase, *GSK3β* glycogen synthase kinase 3β, *LN* lymph node, *MD* moderately differentiated SCC, *PD* poorly differentiated SCC, *SCC* squamous cell carcinoma, *WD* well differentiated SCC.

### Effect of GSK3β inhibition on ESCC cell survival, proliferation and apoptosis

**Figure 2:**
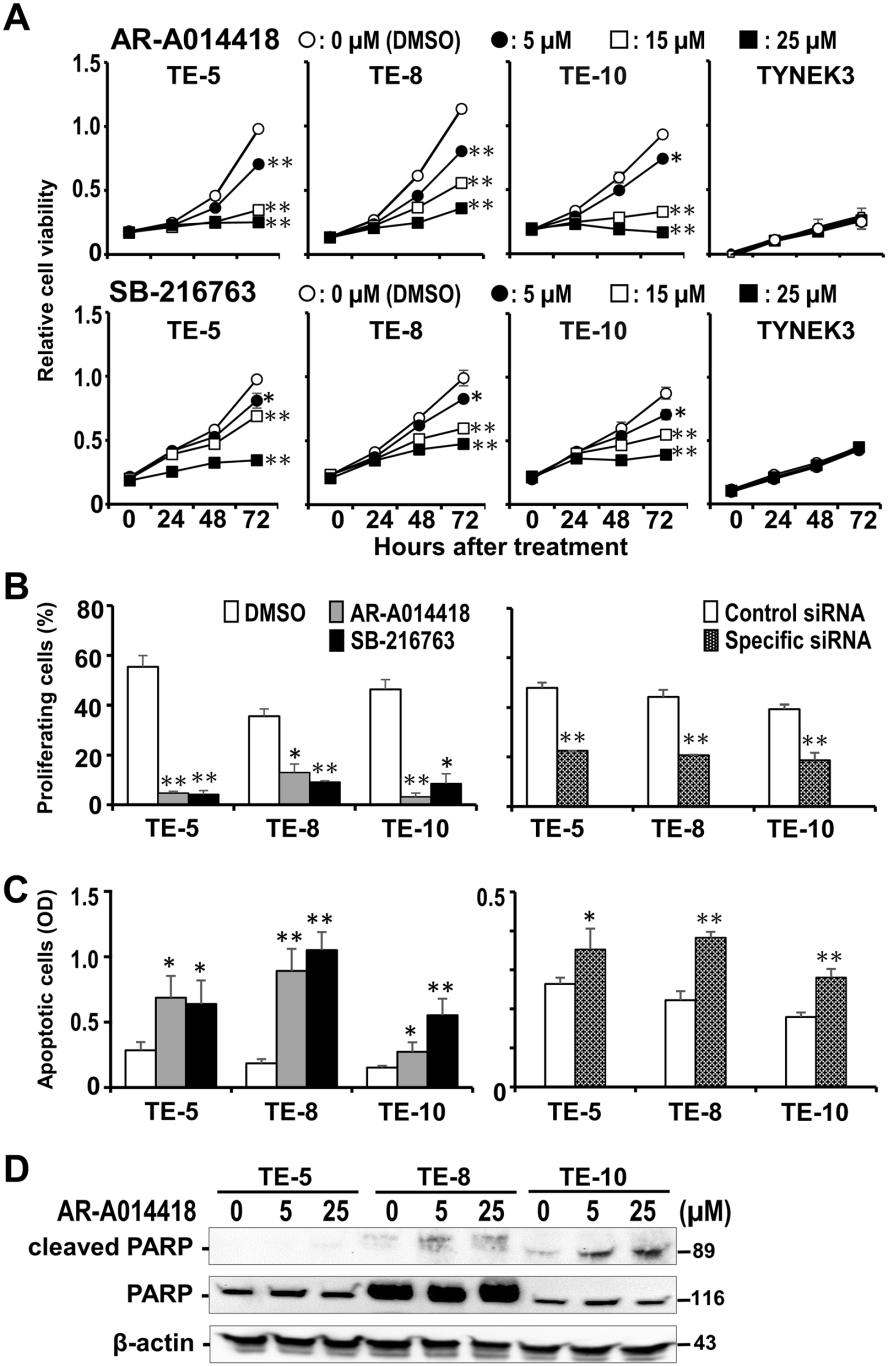
Effects of GSK3β inhibition on cell survival, proliferation and apoptosis in ESCC (TE-5, TE-8, TE-10) and normal esophageal squamous TYNEK-3 cells. (**A**) The respective ESCC cells and TYNEK-3 cells were treated with DMSO or the indicated concentration of AR-A014418 or SB-216763 for the designated times. The relative number of viable cells at each time point was examined by WST-8 assay. Mean values with standard deviations of triplicate experiments were compared between cells treated with DMSO and the indicated GSK3β inhibitor at different concentrations. *P < 0.05, **P < 0.01. (**B**) The incidence of EdU-positive proliferating cells was compared between ESCC cells treated with DMSO (open column), 25 μmol/L AR-A014418 (gray column) and SB-216763 (closed column) for 24 h (left panel), and between the cells transfected with 20 nmol/L control (open column) and GSK3β-specific siRNA (dotted column) for 72 h (right panel). The mean percentages of EdU-positive proliferating cells in 5 fluorescence microscopy fields (shown in Supplementary Information, Fig. S5) were calculated with SDs and statistically compared. (**C**) The mean relative number ± SDs of apoptotic cells measured by DNA fragmentation assay in triplicate was compared between ESCC cells with the same treatment as shown in **B**. (**B**,**C**) * *P* < 0.05; ** *P* < 0.01. (**D**) Western blotting analysis for the amount of PARP and c-PARP in ESCC cells treated with DMSO or the indicated concentrations of AR-A014418 for 24 h. The amount of each protein sample was monitored by the expression of β-actin. Full-length blots for (**D**) are shown in Supplementary Information, Fig. S9.

**Figure 3:**
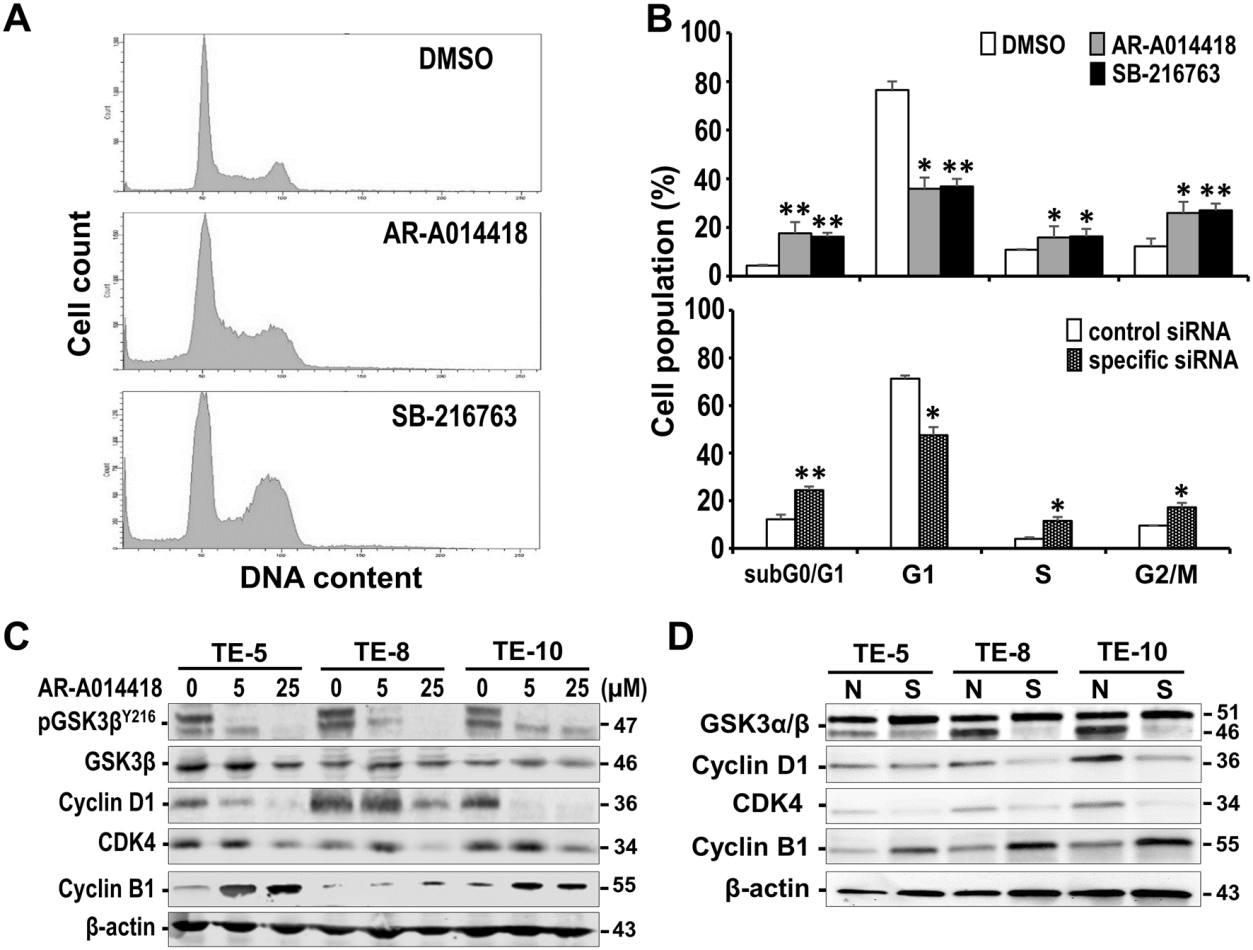
Effects of GSK3β inhibition on the cell cycle profiles and expression of cell cycle-regulating molecules in ESCC cells. (**A**) Representative flow cytometry findings of cell cycle profile of TE-8 cells treated with DMSO, 25 μmol/L AR-A014418 or SB-216763 for 48 h. The data were generated using a FACS Canto II (BD Biosciences). (**B**) Comparison of DNA histograms for each cell cycle fraction of TE-8 cells treated with DMSO (open column), 25 μmol/L AR-A014418 (gray column) or SB-216763 (closed column), or cells transfected for 72 h with non-specific (open column) or GSK3β-specific siRNA (dotted column), respectively. Cellular DNA content was analyzed using FACSDiva software (Version 8.0, BD Bioscience). Data are the mean percentages of cell populations in the respective cell cycle phases with SDs in five separate tests. *P < 0.05; **P < 0.01. (**C**) Western blotting analysis for expression of GSK3β, cyclin D1, CDK4 and cyclin B1 and GSK3β Y216 phosphorylation (pGSK3β^Y216^) in ESCC cells treated with the indicated concentrations of AR-A014418 for 48 h. (**D**) Western blotting analysis for expression of GSK3α/β, cyclin D1, CDK4 and cyclin B1 in ESCC cells transfected with non-specific (N) or GSK3β-specific (S) siRNA, respectively, for 72 h. (**C**, **D**) The amount of each protein sample was monitored by the expression of β-actin**.** Full-length blots for (**C**, **D**) are shown in Supplementary Information, Fig. S9.

To address our hypothesis of a putative tumor-promoting role for GSK3β in ESCC, the biological outcome resulting from GSK3β inhibition was examined in terms of tumor cell survival, proliferation and apoptosis. Treatment with the GSK3β inhibitors (AR-A014418, SB-216763) reduced viability of all ESCC cells in a dose- and time-dependent manner, while sparing normal TYNEK-3 cells (Fig. 2A, Supplementary Information, Fig. S4A). The IC_50_ values of both inhibitors at 48 h after treatment were within the reported pharmacological dose range (1–100 μmol/L) for AR-A014418^30^ and SB-216763^31^. These GSK3β inhibitors decreased the number of EdU-positive proliferating cells (Fig. 2B, Supplementary Information, Fig. S5A) and increased the incidence of apoptosis in ESCC cells (Fig. 2C). Treatment with LY2090314 within the reported pharmacological dose range showed therapeutic effects against ESCC cells that were comparable to AR-A014418 and SB-216763. (Supplementary information, Fig. S6). Induction of apoptosis by GSK3β inhibition was further confirmed by increases in the fraction of c-PARP (Fig. 2D) and the sub-G0/G1 fraction in cell cycle analysis (Fig. 3A,B, Supplementary Information, Fig. S7A). Similar effects were observed in ESCC cells following depletion of GSK3β by siRNA transfection (Fig. 2B,C, Supplementary Information, Fig. S4B,C). These results indicate that ESCC depends on aberrant GSK3β activity for tumor cell survival and proliferation and for evasion of apoptosis, thus implicating this kinase as a potential therapeutic target in ESCC.

### Effects of GSK3β inhibition on cell cycle profile and on cell cycle regulatory molecules

To investigate the mechanism by which GSK3β sustains tumor cell survival and proliferation, we examined the effect of GSK3β inhibition on the cell cycle profile in ESCC cells. FACS analysis showed that treatment of cells with 25 μmol/L GSK3β inhibitors for 48 h decreased the G0/G1-phase fraction, while increasing G2/M-phase and sub-G0/G1 fractions in TE-8, TE-5, and TE-10 cells (Fig. 3A,B, Supplementary Information, Fig. S7A). These effects were reproduced by treatment of the same cells with GSK3β-specific siRNA transfection (Fig. 3B, Supplementary Information, Fig. S7B). Thus, GSK3β inhibition decreased cell entry into the G1 phase and induced apoptosis (corresponding to the sub-G0/G1 fraction), as well as resulting in cell cycle arrest at the G2/M phase. In line with these effects, inhibition of GSK3β decreased the expression of cyclin D1 and CDK4, which as a complex enable cells to enter G1-phase^38^. Inhibition of GSK3β also increased the expression of cyclin B1 (Fig. 3C,D), which is involved in the G2/M phase transition from the S phase^38^. These alterations in cell cycle profiles and in the expression of cell cycle regulatory molecules are consistent with our previous study showing that inhibition of GSK3β induced mitotic catastrophe following G2/M-phase cell cycle arrest in colorectal cancer cells, ultimately resulting in apoptosis^36^. This also suggests the large majority of ESCC cells with GSK3β inhibition in the G2/M phase undergo apoptosis and only cells that survive enter the next round of cell cycle, which is reflected by decreased EdU labeling by GSK3β inhibition (Fig. 2B, Supplementary Figs. S5, S6). Collectively, these results indicate that GSK3β-mediated regulation of cell cycle progression via cyclin D1, CDK4 and cyclin B1 is responsible for tumor cell survival and proliferation in ESCC.

### Effect of GSK3β inhibitors on ESCC xenografts in mice

Prerequisites for clinical translation of a therapy in the investigational phase include its efficacy against the target disease as well as its safety in rodents. In this study, we tested the efficacy of GSK3β inhibitors against TE-8 cell xenograft tumors in mice. The inhibitors included AR-A014418 and LY2090314, with the latter having previously been tested in clinical trials^39,40^. Prior to the animal experiments, cell survival assays showed a dose- and time-dependent effect of LY2090314 against TE-8 cells, with IC_50_ = 2.18 μmol/L after treatment for 72 h (Supplementary Information, Fig. S6). Based on the IC_50_ concentration for LY2090314 used in vitro in the present study and in previous preclinical^41^ and clinical studies^39,40^, we used doses of 1 mg/kg and 2.5 mg/kg body weight in the xenograft study.

**Figure 4:**
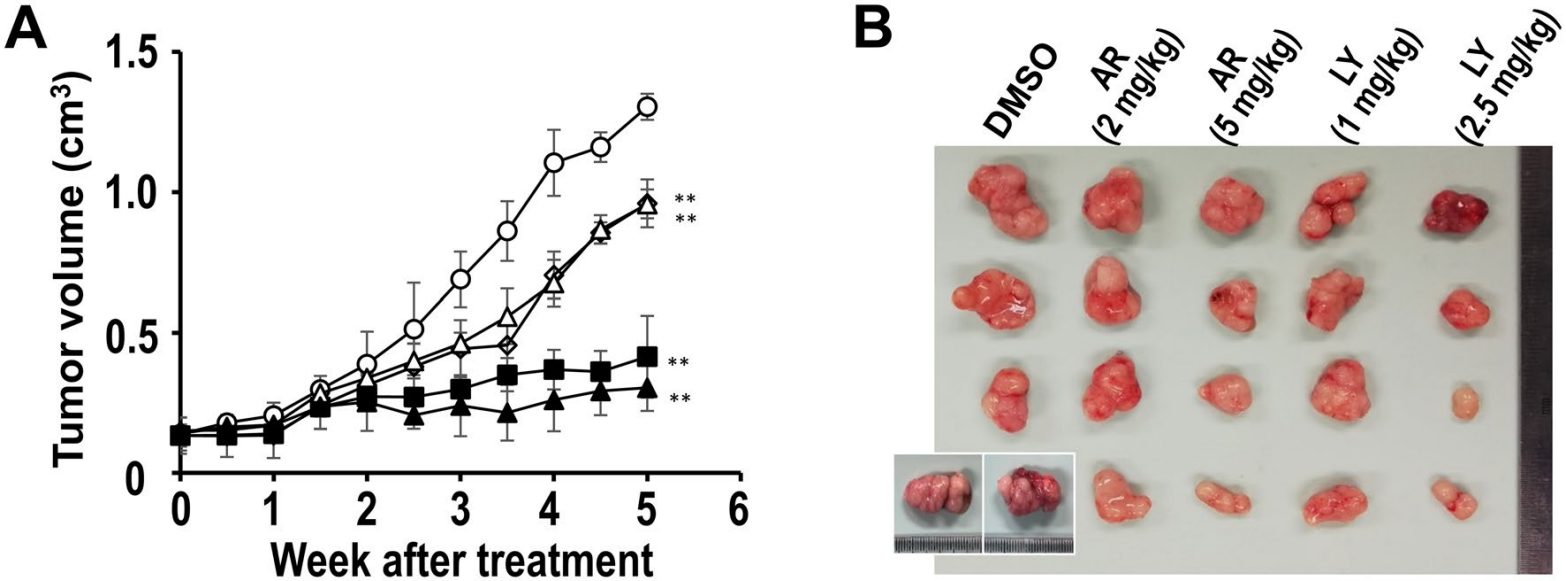
Effect of GSK3β inhibitors on proliferation of ESCC cell xenograft tumors in mice. (**A**) Time course of the effects of DMSO (open circle), AR-A014418 (2 mg/kg body weight, open square; 5 mg/kg body weight, closed square), and LY2090314 (1 mg/kg body weight, open triangle; 2.5 mg/kg body weight, closed triangle) on tumor size of TE-8 cell xenografts in mice. (**B**) Gross appearance of xenograft tumors removed at autopsy from mice after 5 weeks of treatment with different doses of AR-A014418 (AR) or LY2090314 (LY). The left lower insets show the tumors removed from two mice 4 weeks after treatment with DMSO following animal experimentation ethics guidelines as described in the “Results”. **P < 0.01.

Compared to DMSO, treatment of mice with AR-A014418 and LY2090314 significantly reduced the xenograft tumor volume in a dose- and time-dependent manner (Fig. 4A,B). Following treatment for 4 weeks, we sacrificed two DMSO-treated mice that showed a body weight loss of < 18 g (Supplementary Information, Fig. S1B) according to the animal ethics guidelines. Throughout the scheduled treatment period, no obvious detrimental effects or adverse events were observed in mice undergoing treatment with the GSK3β inhibitors. At necropsy, gross observation and histologic examination showed no pathologic findings and no primary or metastatic tumors in the vital organs including lungs, liver, pancreas and kidneys of all mice (not shown).

**Figure 5:**
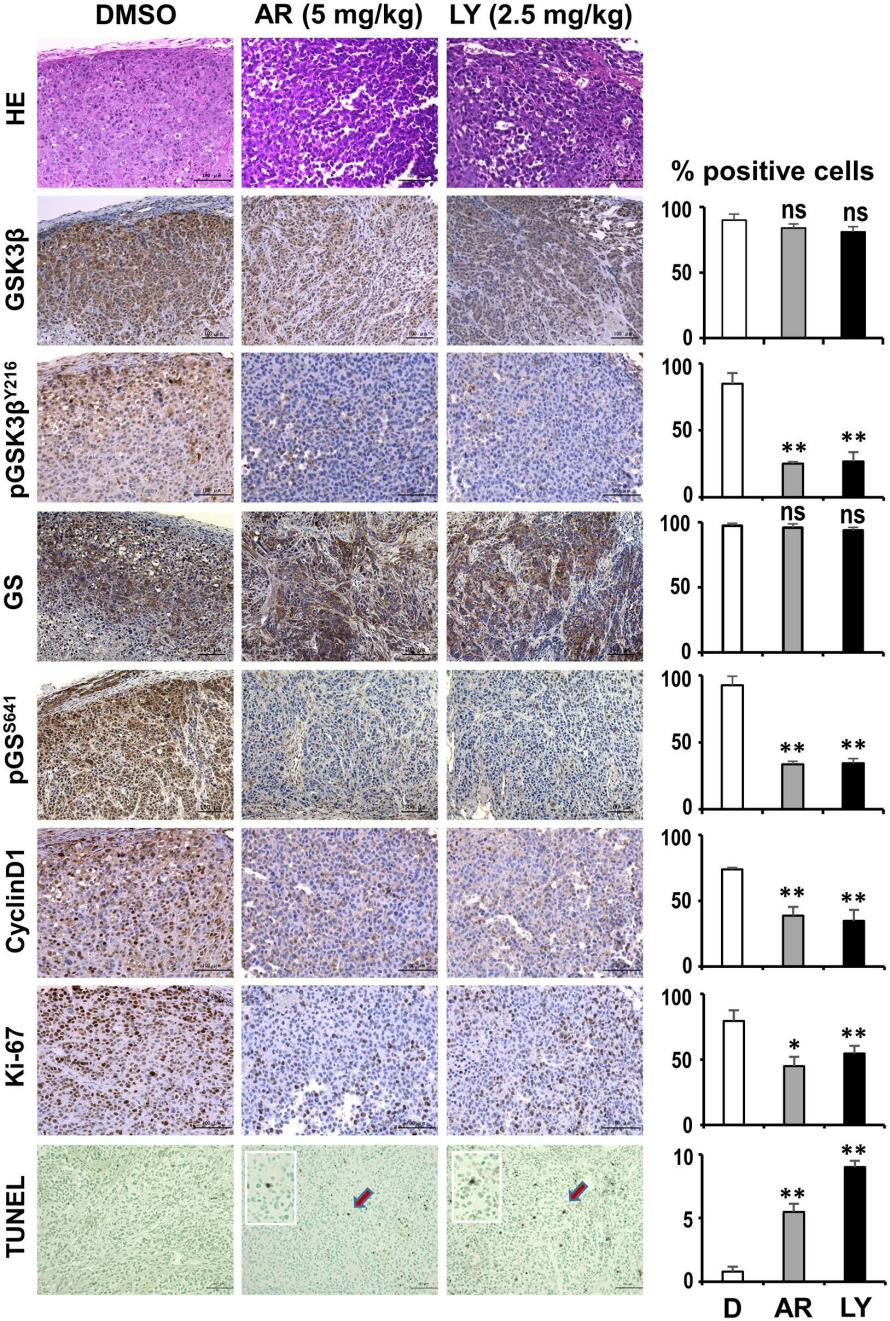
Representative histological, immunohistochemical and histochemical findings of xenograft tumors from mice treated with DMSO, 5 mg/kg body weight AR-A014418 (AR) or 2.5 mg/kg body weight LY2090314 (LY). Serial paraffin sections of the respective tumors were stained with hematoxylin and eosin (HE), immunostained for GSK3β, pGSK3β^Y216^, GS, pGS^S641^, cyclin D1 and Ki-67, and histochemically stained by the TUNEL method. A scale bar in each panel indicates 100 μm. Histological, immunohistochemical and histochemical images were captured using Keyence BZ-X700 Analyzer (Version 1.3). The graphs on the right side show statistical comparison of the mean percentages with SDs of tumor cells positive for the corresponding molecules, and TUNEL results for xenograft tumors from mice treated with DMSO, AR or LY. *P < 0.05; **P < 0.01.

We compared the level of pGSK3β^Y216^ (active form) in the tumors of mice treated with DMSO or with the GSK3β inhibitors. Immunohistochemistry showed significantly lower levels of pGSK3β^Y216^ in the tumors of inhibitor-treated mice (Fig. 5), consistent with the results from Western blotting (Supplementary Information, Fig. S8A). These findings indicate that decreased pGSK3β^Y216^ level is probably a consequence of the GSK3β inhibitors acting against GSK3β in tumor cells in mice, although the primary mechanism of action of these inhibitors is to compete with ATP for the ATP-binding pocket in GSK3β^30,34^. Similar to ESCC cell cultures (Figs. 2B–D, 3C, Supplementary Information, Fig. S5, S6), the GSK3β inhibitors significantly reduced tumor cell proliferation (Ki-67-positive cells), pGS^S641^ level and cyclin D1 expression, while inducing apoptosis (TUNEL- and c-PARP-positive cells) (Fig. 5, Supplementary Information, Fig. S8B).

## Discussion

Different treatment options for ESCC depend on the tumor stage and on patient tolerability to the treament^2,5^. Non-invasive and minimally invasive tumors with little risk of lymph node metastasis are rare and are amenable to endoscopic resection, radiofrequency ablation and photodynamic therapy. Patients with locally invasive ESCCs but no distant metastasis undergo transthoracic esophagectomy with lymphadenectomy. Patients with locally advanced and/or metastatic ESCC account for the vast majority of patients and require treatment with various combinations of chemotherapy, radiation and surgery. Recently, biological agents that target epidermal growth factor receptor (e.g., gefitinib)^42^ and programmed cell death protein 1 (PD-1) pathway (e.g., nivolumab)^43^ have been tested for advanced ESCC patients who are refractory to multipronged therapy. However, the overall efficacy of these therapies has been low and the overall 5-year survival rate of ESCC patients is between 15 to 25%. ESCC is the 6th leading cause of cancer-related mortality worldwide^3–5,44^ and the dismal outlook of this disease has led us to investigate the putative tumor-promoting role of GSK3β as a new therapeutic target in ESCC.

Based on its physiological functions against major proto-oncogenic pathways driven by Wnt/β-catenin, hedgehog, notch and c-Myc signaling, as well as against epithelial-to-mesenchymal transition, GSK3β has long been recognized to suppress tumor development and progression^18,22^. Several previous studies on the tumor-suppressive roles of GSK3β in various oncogenic pathways showed that it was inactivated mostly through S9 phosphorylation. However, there was no evidence that active GSK3β suppresses the development and progression of tumors by disrupting the major proto-oncogenic (e.g., Wnt/β-catenin) pathways (reviewed in Ref.^20^). It was reported that Axl oncoprotein promotes the development and progression of ESCC via inactivation of GSK3β and activation of the NF-κB pathway^45^. However, this study did not measure the basal levels of expression and/or activity of GSK3β in the tumor cells, nor did it investigate the direct effects of GSK3β inhibition on tumor cell survival, proliferation and apoptosis. In contrast, many previous studies have demonstrated direct tumor-promoting roles of GSK3β in at least 25 different cancer types (reviewed in Ref.^23^).

In the present study, we found that expression and activity of GSK3β in ESCC cell lines and primary tumors was higher than in normal esophageal squamous mucosal cells and tissues. Consistent with previous studies by our group and others (reviewed in Refs.^18–21,23^), inhibition of GSK3β attenuated tumor cell survival and proliferation and induced apoptosis in ESCC cells and in xenograft tumors. Previous studies showed a similar therapeutic effect of lithium, a non-specific and ATP-non-competitive GSK3 inhibitor, against ESCC^46,47^. Here we showed no harmful effects of pharmacological GSK3β inhibitors on normal human esophageal squamous cells and on the esophagus and vital organs of mice. Taken together, the present study reinforces the notion that GSK3β is a potential therapeutic target in ESCC, thereby including this as another tumor type susceptible to GSK3β-targeted therapy^19,21^.

Uncontrolled cell cycle progression is one of the biological hallmarks of cancer. Novel cancer therapeutics include agents that target microtubule dynamics and inhibitors of mitotic kinases such as cyclin-dependent kinases (CDKs)^38,48^. In the present study, the therapeutic effects of GSK3β inhibition in ESCC cells included cell cycle arrest at G0/G1- and G2/M-phase, decreased expression of cyclin D1 and CDK4, and increased cyclin B1 expression. We and others have previously shown that inhibition of GSK3β in human colon and breast cancer cells induced mitotic catastrophe by disturbing centrosome dynamics and the assembly of spindle apparatus, with the cells ultimately undergoing apoptosis^36,49^. Overall, our results suggest that ESCC cells depend on deregulated GSK3β for their survival and proliferation via cyclin D1 and CDK4-mediated G0/G1-phase cell cycle progression and G2/M-phase cell cycle transition. As reviewed recently by our group^23^, the tumor-promoting roles of GSK3β involve diverse arrays of pro-oncogenic pathways, suggesting a need for future studies to clarify the distinct biological mechanism(s) by which GSK3β participates in the progression of ESCC.

One of the earliest biochemical changes during ESC carcinogenesis is the disappearance of glycogen in transformed cells. Loss of glycogen in the early stages of squamous cell carcinogenesis was first observed in uterine cervical squamous mucosa in 1933^50^. This fundamental biochemical characteristic enables the Schiller test and Lugol’s dye endoscopy for early diagnosis of squamous cell carcinoma in the uterine cervix and esophagus^2,50^. However, the biological mechanism for glycogen loss is not yet fully understood. In the present study we observed the level of pGS^S641^ was higher in ESCC cells and primary tumors than in normal TYNEK-3 cells and normal squamous mucosa, indicating the inactivation of GS in ESCC. This coincided with increased pGSK3β^Y216^ (active form) and decreased pGSK3β^S9^ (inactive form) levels in ESCC cells and tumors. These observations may explain the depletion of glycogen in ESCC cells.

Among the biological hallmark characteristics of cancer, aberrant glycolysis as represented by the Warburg effect is the strongest and most critical selective pressure for cellular transformation and malignant evolution in the majority of cancer types^51–53^, including ESCC^54^. The substrates of GSK3β include a number of key metabolic enzymes, suggesting this kinase could have broad control over various physiological and pathological metabolic pathways^15,16^. The primary role of GSK3β is to control GS activity via S641 phosphorylation, thus acting at the bifurcation between glycogen synthesis and glycolysis, the two major pathways of glucose/glycogen metabolism. Preliminary findings from the present study showed that intracellular constitutive levels of glycogen in ESCC cells were significantly lower than in normal TYNEK-3 cells, but were restored following treatment with GSK3β inhibitor. Accordingly, our observations suggest that deregulated GSK3β may shift ESCC cell metabolism from glycogenesis to the glycolytic pathway. The latter fuels the synthesis of biomacromolecules (nucleic acids, amino acids, lipids) and energy (ATP) production, both of which are mandatory for sustained cell survival and proliferation. Our results also provide novel insight into how glucose metabolism is reprogrammed in cancer cells^55^.

## Supplementary information

Supplementary Information.

## Abbreviations

ABC: Avidin–biotin–peroxidase complex
CDK: Cyclin-dependent kinase
cPARP: Cleaved PARP
DAPI: 4′,6-Diamidino-2-phenylindole
DMSO: Dimethyl sulfoxide
dUTP: 2′-Deoxyuridine-5′-triphosphate
EdU: 5-Ethynyl-2′-deoxyuridine
FACS: Fluorescence-activated cell sorting
GS: Glycogen synthase
GSK3β: Glycogen synthase kinase-3β
IHC: Immunohistochemistry
PARP: Poly [ADP-ribose] polymerase
PBS: Phosphate buffered saline
pGSK3β^S9^: GSK3β phosphorylated in S9 residue
pGSK3β^Y216^: GSK3β phosphorylated in Y216 residue
pGS^S641^: GS phosphorylated at S641 residue
PI: Propidium iodide
S: Serine
SD(s): Standard deviation(s)
siRNA: Small interfering RNA
TCGA: The Cancer Genome Atlas
TUNEL: Terminal deoxynucleotidyl transferase-mediated dUTP nick end labeling
Y: Tyrosine
