# Supplementary material for: Potential therapeutic effect of targeting glycogen synthase kinase 3β in esophageal squamous cell carcinoma
Source: Sci Rep. 2020 Jul 16;10:11807. doi: 10.1038/s41598-020-68713-9 (PMC7367341; doi:10.1038/s41598-020-68713-9)
Supplement: Supplementary file 1 — Supplementary Information. [file 41598_2020_68713_MOESM1_ESM.docx]

**Supplementary Information**

**Potential therapeutic effect of targeting glycogen synthase kinase 3β in esophageal squamous cell carcinoma**

Dilireba Bolidong^1^, Takahiro Domoto^1^, Masahiro Uehara^1^, Hemragul Sabit^3^, Tomoyuki Okumura^6^, Yoshio Endo^2^, Mitsutoshi Nakada^3^, Itasu Ninomiya^4^, Tomoharu Miyashita^4,7^, Richard W. Wong^5^, Toshinari Minamoto^1^

¹Division of Translational and Clinical Oncology and ^2^Central Research Resource Branch, Cancer Research Institute; Departments of ^3^Neurosurgery and ^4^Gastroenterological Surgery, Graduate School of Medical Science; ^5^WPI Nano Life Science Institute, Kanazawa University; ^6^Department of Surgery and Science, Graduate School of Medicine and Pharmaceutical Sciences, University of Toyama;

^7^Department of Surgical Oncology, Kanazawa Medical University Hospital, Ishikawa, Japan.

**Corresponding author:** Toshinari Minamoto, MD, PhD, Division of Translational and Clinical Oncology, Cancer Research Institute, Kanazawa University, 13-1 Takara-machi, Kanazawa 920-0934, Japan. Phone: 81-76-265-2792; FAX: 81-76-234-4529; e-mail: [minamoto@staff.kanazawa-u.ac.jp](mailto:minamoto@staff.kanazawa-u.ac.jp)

**Supplementary Figure S1.** The design and protocol of the animal experiment is shown in **(A)** and the body weight of mice in **(B)**.

**(A)** At week 0, TE-8 cells (1 × 10^6^) were inoculated subcutaneously. The mice were randomly assigned to five groups at 3 weeks after inoculation. The respective groups of mice were treated by tri-weekly intraperitoneal (i.p.) injections of DMSO, AR-A014418 (AR) and LY2090314 (LY) at the indicated doses. After 5 weeks of treatment, all mice were euthanized for necropsy. **(B)** Time course of the changes in mean body weight of mice in the respective groups with different treatments. *At 4 weeks after treatment, two mice in the DMSO-treated (control) group were euthanized because of their body weight loss (less than 18 gr).

**Supplementary Figure S2.** Comparative analysis for expression and phosphorylation of glycogen synthase (GS) in human ESCC cells and normal esophageal squamous epithelial (TYNEK-3) cells and in normal squamous mucosa and primary tumors of ESCC patients.

**(A)** Expression of GS and of its S641 phosphorylation (pGS^S641^, inactive form) in the cells was examined by Western blotting. β-actin expression was examined as a loading control in each sample. **(B)** The left panel showed constitutive intracellular glycogen concentration in normal TYNEK-3 cells and ESCC cell lines. The right panel showed the effect of GSK3β inhibitors on intracellular glycogen concentration in TE-5, TE-8 and TE-10 cells. **(C)** Representative findings for the expression of GS and its S641 phosphorylation (pGS^S641^) in the primary tumor and in corresponding normal squamous mucosa of ESCC patients. The scale bar indicates 100 μm in length. Immunohistochemical images were captured using Keyence BZ-X700 Analyzer (Version 1.3). The right two graphs generated using GraphPad Prism 5.0 (GraphPad Software, Inc. CA) show statistical comparison of the immunohistochemistry (IHC) scores of GS and pGS^S641^ between the primary tumor (T) and normal mucosa (N) of ESCC patients. * *P* < 0.05; ** *P* < 0.01. Full-length blots for **(A)** are shown in Supplementary Information, Fig. S9.

**Supplementary Figure S3.** Comparison of tumor GSK3β mRNA levels with clinical parameters (age, gender, body weight, smoking habits), tumor grade and stage of ESCC based on TCGA Database.

These data were generated using the analysis tool UALCAN (http://ualcan.path.uab.edu/).^33^

**Supplementary Figure S4.** Effects of GSK3β inhibition on cell survival in ESCC cells.

**(A)** The respective ESCC cells were treated with DMSO or the indicated concentrations of AR-A014418 or SB-216763 for the designated times. The relative number of viable cells at each time point was examined by WST-8 assay. The mean values with standard deviations of triplicate experiments were statistically compared between the same cells treated with DMSO and the indicated GSK3β inhibitor at different concentrations. * *P* < 0.05, ** *P* < 0.01. **(B)** Western blot analysis comparing the levels of expression of GSK3α and GSK3β between the respective cells transfected with non-specific (N) or GSK3β-specific (S) siRNA. **(C)** The mean relative number with SDs of viable cells was statistically compared between cells transfected with non-specific (control) and GSK3β-specific siRNA, respectively. ** *P* < 0.01. Full-length blots for **(B)** are shown in Supplementary Information, Fig. S9.

**Supplementary Figure S5.** Representative fluorescence microscopy findings of ESCC cells for the measurement of cell proliferation. Respective ESCC cells were treated with DMSO, 25 µmol/L AR-A014418 or SB-216763 for 48 hours **(A)**, or transfected with nonspecific (control) or GSK3β-specific siRNA **(B)**.

The cells were labeled with EdU (red) and counterstained for nuclei with Hoechst 33340 (blue). The scale bar in each panel represents 100 μm. These fluorescence microscopy images were captured using Keyence BZ-X700 Analyzer (Version 1.3). The number and percentage of nuclear EdU-positive cells among the total number of nuclei was scored and shown below each panel.

**Supplementary Figure S6.** Effect of LY2090314 on cell survival, proliferation and apoptosis in the ESCC cells.

**(A)** The respective ESCC cells were treated with DMSO or the indicated concentrations of LY2090314 for the designated times. The relative number of viable cells at each time point was examined by WST-8 assay. The mean values with SDs of triplicate experiments were statistically compared between the same cells treated with DMSO and LY2090314 at different concentrations. **(B)** Fluorescence microscopy findings of ESCC cells sequentially stained with EdU (red) and nuclear counterstained with Hoechst 33340 (blue) following treatment with DMSO or 3 μmol/L LY2090314 for 48 hours. The scale bar in each panel indicates 100 μm. These fluorescence microscopy images were captured using Keyence BZ-X700 Analyzer (Version 1.3). The number and percentage of nuclear EdU-positive cells among the total number of nuclei was scored and shown below each panel. **(C)** The mean percentages of EdU-positive proliferating cells in 5 fluorescence microscopy fields were calculated with SDs and statistically compared between cells treated with DMSO or LY2090314. **(D)** The mean relative number ± SDs of apoptotic cells measured by DNA fragmentation assay in triplicates was compared between ESCC cells treated with DMSO or LY2090314. **(A, C, D)** * *P* < 0.05; ** *P* < 0.01.

**Supplementary Figure S7.** Effect of GSK3β inhibition on the cell cycle profile of ESCC cells. TE-5 and TE-10 cells were treated with DMSO, 25 μmol/L AR-A014418 (AR) or SB-216763 (SB) for 48 hours **(A)**, or transfected with non-specific (control) or GSK3β-specific siRNA, respectively, for 72 hours **(B)**.

Cell cycle profiles of the respective cells were analyzed by flow cytometry using a FACS Canto II (BD Biosciences). Cellular DNA content was analyzed using FACS Diva software (Version 8.0, BD Bioscience) as shown in Fig. 3. * *P* < 0.05; ** *P* < 0.01.

**Supplementary Figure S8.** Effects of GSK3β inhibitors on the biological characteristics of ESCC xenograft tumors in mice.

**(A)** Western blot analysis for the expression of cyclin D1 and GSK3β and for its Y216 phosphorylation (pGSK3β^Y216^) in TE-8 cell xenograft tumors in mice treated with DMSO (n=4) or with the indicated concentrations of AR-A014418 (AR; n=4) and LY2090314 (LY; n=4). Expression of β-actin was monitored as a loading control. **(B)** Representative fluorescence microscopy findings of xenograft tumors in mice for measurement of apoptotic cells. Serial sections of tumors from mice treated with DMSO, AR-A01448 (AR) or LY2090314 (LY) were stained with HE. The sections were also immunofluorescently stained for c-PARP (red) and PARP (green), and the nuclei counterstained with DAPI (blue). The scale bar in each panel represents 100 μm. These histological and immunofluorescence microscopy images were captured using Keyence BZ-X700 Analyzer (Version 1.3). Merged images for c-PARP and DAPI are shown at the bottom. Full-length blots for (A) are shown in Supplementary Information, Fig. S9.

**Supplementary Figure S9.** The full-length blots of Fig. 1A, 2D, 3C, 3D, and Supplementary Information, Fig. 2A, 4B and 8A.

**Supplementary Figure S9 (continued).**

**Supplementary Figure S9 (continued).**

**Supplementary Figure S9 (continued).**
